# A mouse model for fucosidosis recapitulates storage pathology and neurological features of the milder form of the human disease

**DOI:** 10.1242/dmm.025122

**Published:** 2016-09-01

**Authors:** Heike Wolf, Markus Damme, Stijn Stroobants, Rudi D'Hooge, Hans Christian Beck, Irm Hermans-Borgmeyer, Renate Lüllmann-Rauch, Thomas Dierks, Torben Lübke

**Affiliations:** 1 Biochemistry I, Department of Chemistry, Bielefeld University, Bielefeld D-33615, Germany; 2 Biochemical Institute, University of Kiel, Kiel D-24098, Germany; 3 Laboratory of Biological Psychology, University of Leuven, Leuven B-3000, Belgium; 4 Department of Biochemistry and Pharmacology, Centre for Clinical Proteomics, Odense University Hospital, Odense DK-5000, Denmark; 5 ZMNH, University Medical Center Hamburg-Eppendorf, Hamburg D-20246, Germany; 6 Department of Anatomy, University of Kiel, Kiel D-24098, Germany

**Keywords:** Fucosidosis, Neurodegeneration, Lysosomal storage disorder

## Abstract

Fucosidosis is a rare lysosomal storage disorder caused by the inherited deficiency of the lysosomal hydrolase α-L-fucosidase, which leads to an impaired degradation of fucosylated glycoconjugates. Here, we report the generation of a fucosidosis mouse model, in which the gene for lysosomal α-L-fucosidase (*Fuca1*) was disrupted by gene targeting. Homozygous knockout mice completely lack α-L-fucosidase activity in all tested organs leading to highly elevated amounts of the core-fucosylated glycoasparagine Fuc(α1,6)-GlcNAc(β1-N)-Asn and, to a lesser extent, other fucosylated glycoasparagines, which all were also partially excreted in urine. Lysosomal storage pathology was observed in many visceral organs, such as in the liver, kidney, spleen and bladder, as well as in the central nervous system (CNS). On the cellular level, storage was characterized by membrane-limited cytoplasmic vacuoles primarily containing water-soluble storage material. In the CNS, cellular alterations included enlargement of the lysosomal compartment in various cell types, accumulation of secondary storage material and neuroinflammation, as well as a progressive loss of Purkinje cells combined with astrogliosis leading to psychomotor and memory deficits. Our results demonstrate that this new fucosidosis mouse model resembles the human disease and thus will help to unravel underlying pathological processes. Moreover, this model could be utilized to establish diagnostic and therapeutic strategies for fucosidosis.

## Abstract (precis)

**Summary:** The fucosidosis mouse model aids understanding of general pathophysiological mechanisms underlying α-L-fucosidase deficiency and is urgently required to establish therapeutic approaches.

## INTRODUCTION

Fucosidosis (OMIM ID 230000) is an ultra-rare neurodegenerative and progressive lysosomal storage disease (LSD) in humans that is caused by a defect in lysosomal α-L-fucosidase [ExPASy enzyme (EC) ID 3.2.1.51] (Van Hoof and Hers, 1968). The disease is inherited in an autosomal-recessive manner and no more than ∼120 cases worldwide have been reported since it was initially described in the late 1960s (Durand et al., 1968; Willems et al., 1991). Some studies, however, indicate that its incidence is much higher than reported, particularly in some regions of Southern Italy, some parts of Cuba, and Tunisia, as well as in some populations of Mexican-Indian origin in Arizona and Colorado of the United States (Ben Turkia et al., 2008; Menéndez-Sainz et al., 2012; Willems et al., 1991).

On the basis of the clinical course, fucosidosis has been divided into a severe infantile fast-progressing form (type 1) and a milder form (type 2), although the disease often presents with a continuum of an entire set of clinical features. Fucosidosis is dominated by neurological symptoms like progressive mental and motor deterioration, and seizures, but it is often accompanied by coarse facial features, growth retardation, dysostosis multiplex, angiokeratoma, visceromegaly and a broad range of further symptoms. Half of the affected individuals die before 10 years of age (Willems et al., 1991). So far, at least 29 different mutations in the *FUCA1* gene on chromosome 1p34 of fucosidosis individuals have been identified, most of them in homozygous form due to high consanguinity (Malm et al., 2012). Like many other LSDs, fucosidosis lacks a clear genotype-phenotype relationship, and the same homozygous *FUCA1* mutation can lead to either the type-1 or the type-2 phenotype (Willems et al., 1991). Of note, there is a second fucosidase, called plasma α-L-fucosidase (Eiberg et al., 1984), which is encoded by the *FUCA2* gene. So far, it is unclear whether or not this enzyme might contribute to α-L-fucosidase activity and therefore could represent a disease modifier.

Biochemically, fucosidosis is characterized by impaired lysosomal degradation of fucosylated glycoproteins and glycolipids as the disease-causing α-L-fucosidase catalyzes the cleavage of α1,2-, α1,3-, α1,4- as well as α1,6-linked fucosyl residues within the entire set of glycoconjugates (Johnson and Alhadeff, 1991; Shoarinejad et al., 1993). Thus, a considerable number of more than 20 fucosylated substrates are known to accumulate in great amounts in various tissues which, as a consequence, are also excreted in the urine of affected individuals (Michalski and Klein, 1999). Beside oligosaccharides and glycolipids, which are common storage products of glycoproteinoses, the vast majority of storage material comprises fucosylated glycoproteins and glycoasparagines (Strecker et al., 1978). These compounds are exclusively detected in fucosidosis and hence can be used as diagnostic biomarkers. In liver, brain, pancreas and skin of fucosidosis individuals, severely affected cell types often show extensive vacuolation with a foam-cell-like appearance. Although most cell types show empty vacuoles, indicating storage of water soluble material, the vacuoles in some cell types also include granular or lamellar electron-dense structures, as detected by electron microscopy, indicating more heterogeneous storage material than is known from other LSDs (Willems et al., 1991).

To date, no general treatment for fucosidosis is available. Very few individuals have been successfully treated with bone marrow transplantation (BMT) but there has been at least some neurological improvement in some cases (Krivit et al., 1999); however, graft-versus-host complications also occurred (Miano et al., 2001). A dog model in English Springer spaniels was characterized a long time ago, which closely resembles the human disease (Abraham et al., 1984; Fletcher et al., 2014; Fletcher and Taylor, 2016; Hartley et al., 1982; Kondagari et al., 2011b), and thus was used to establish BMT (Taylor et al., 1992, 1986) as well as enzyme replacement therapy (ERT) (Kondagari et al., 2015, 2011a). Moreover, a domestic shorthair cat model lacking fucosidase activity has been reported, and shows cerebellar dysfunction and storage pathology (Arrol et al., 2011).

In this study, we establish a knockout mouse model for fucosidosis by using a gene replacement strategy and demonstrate that this mouse model is an easy to manage model system in order to understand the mechanisms of disease progression, to identify putative biomarkers for reliable diagnosis and to address therapeutic strategies such as ERT.

## RESULTS

### Generation of a fucosidosis mouse model and confirmation of *Fuca1* inactivation

In order to understand the pathological mechanisms underlying fucosidosis, we generated a constitutive knockout mouse model by inserting the *Escherichia* *coli* neomycin phosphotransferase I *(nptI)* gene into exon 1 of the *Fuca1* gene that encodes lysosomal α-L-fucosidase (Fig. S1A). Correct homologous recombination of the gene-targeting construct was confirmed by performing PCR amplification with genomic DNA, resulting in a 4.1-kb fragment for the wild-type allele and a 5.3-kb fragment for the knockout allele (Fig. S1B, upper panel), and subsequent sequencing of the PCR products. Routine genotyping was performed with a multiplex PCR using an *nptI*-specific primer for detection of the knockout allele and a primer that bound to exon 1 for detection of the wild-type allele (Fig. S1B, lower panel). Transcriptional inactivation of *Fuca1* was initially validated in several tissues by performing quantitative real-time PCR (qPCR), and revealed some residual *Fuca1* mRNA in spleen and brain but not in liver and kidney (Fig. S1C).

**Fig. 1.**
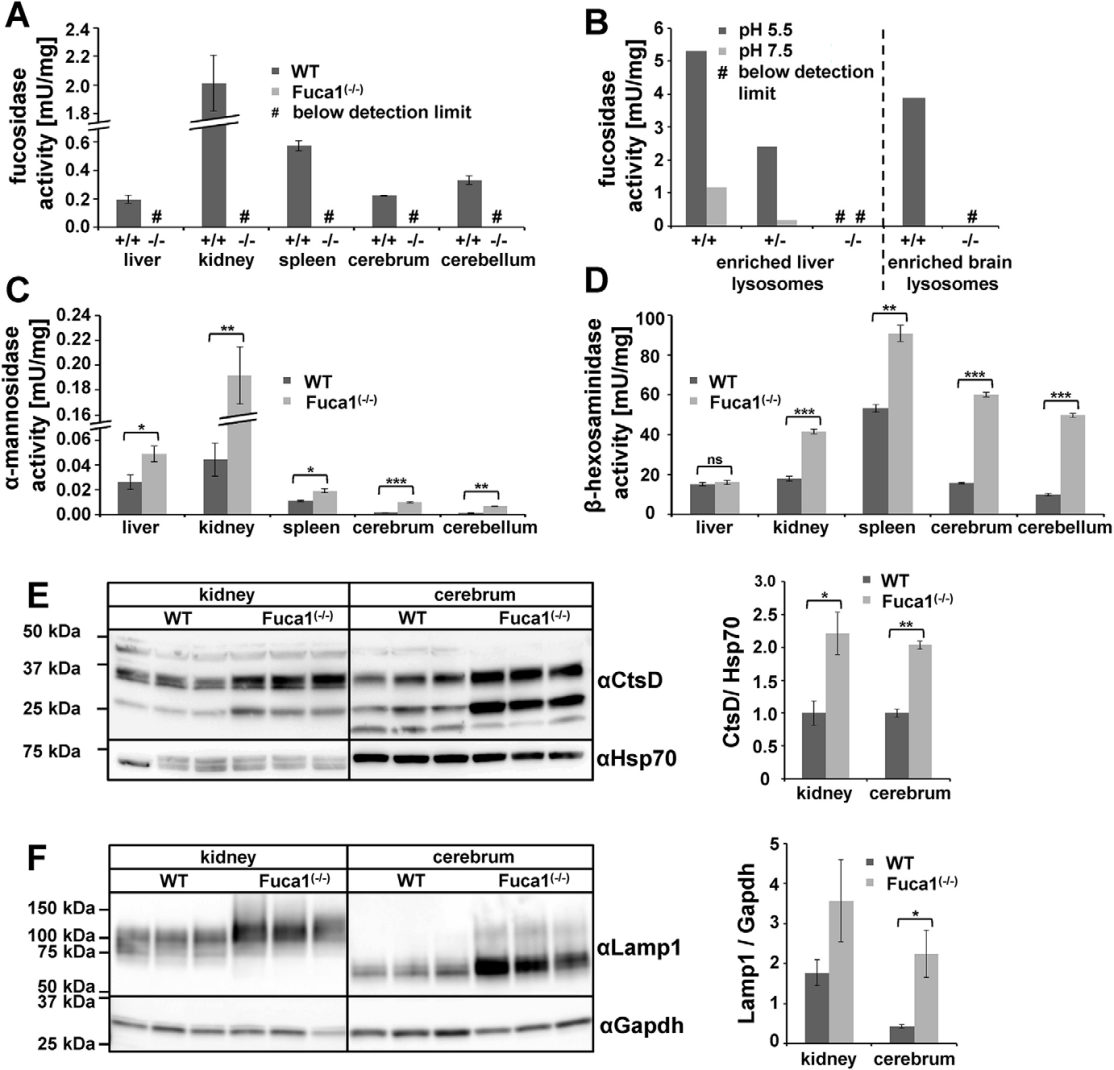
***Fuca1* deficiency validation and expression of lysosomal enzymes.** (A) Specific α-L-fucosidase activity was measured at pH 5.5 in several tissue homogenates of 5-month-old wild-type (WT, +/+) and *Fuca1^−/−^* mice. No α-L-fucosidase activity was detected in *Fuca1*-deficient (−/−) tissues (mean±s.d., *n*=3). (B) Specific α-L-fucosidase activity was determined in isolated liver lysosomes of wild-type, *Fuca1^+/−^* and *Fuca1^−/−^* mice at pH 5.5 and 7.5. Brain lysosomes from wild-type and *Fuca1^−/−^* mice were analyzed at pH 5.5. No α-L-fucosidase activity was detected in lysosomes from knockout mice. (C,D) Enzyme activity of the lysosomal glycosidases α-mannosidase and β-hexosaminidase are increased in several tissue homogenates of 5-month-old *Fuca1-*deficient mice (mean+s.d., *n*=3). (E,F) Immunoblotting of wild-type and *Fuca1^−/−^* tissue homogenates from kidney and cerebrum (5-month-old mice) revealed an increase in cathepsin D (αCtsD) and in lysosome-associated membrane protein 1 (αLamp1) expression (mean±s.d., *n*=3). The different molecular weights of Lamp1 are attributed to organ-specific glycosylation. *P<0.05, **P<0.005, ***P<0.0005 and ns, not significant (two-tailed *t*-test).

To exclude that these residual *Fuca1* transcripts translate into functional protein, tissue homogenates as well as liver-derived and brain-derived lysosome-enriched fractions (∼20-30-fold enriched in lysosomal hydrolases) were analyzed for α-L-fucosidase activity using the artificial pseudosubstrate 4-methylumbelliferyl-α-L-fucopyranoside (4-MU-Fuc). α-L-fucosidase activity was totally absent from all tested tissue homogenates derived from *Fuca1*-deficient mice, as assayed at pH 5.5 (Fig. 1A). Kidney homogenates were assayed over the entire pH range from 4 to 8 and also showed no detectable α-L-fucosidase activity (Fig. S1D).

Moreover, lysosome-enriched fractions from liver exhibited high α-L-fucosidase activity in wild-type samples (∼ 25-fold higher than liver homogenates), whereas such lysosomal fractions from knockout mice were totally devoid of activity both at acidic and neutral pH (Fig. 1B, left). The lysosomal glycosidase β-hexosaminidase showed comparable specific activities in both genotypes (data not shown). Similarly, α-L-fucosidase activity was not detectable in a lysosome-enriched fraction from knockout brain (Fig. 1B, right), whereas β-hexosaminidase activity was even elevated, indicating enrichment of lysosomes, in comparison to fractions from wild-type mice (Fig. S1E,F). Taken together, these results on genomic, transcript and enzyme levels unambiguously confirm *Fuca1* deficiency of our mouse model and suggest that it can be used as a reliable fucosidosis animal model.

### Regulation of other lysosomal hydrolases involved in glycoprotein degradation

We next determined transcript levels and enzymatic activities of several other lysosomal hydrolases, which are involved in glycoprotein degradation, as well as the expression level of the lysosomal membrane protein Lamp1. qPCR analyses of mRNA levels from cerebrum of 5-month-old wild-type and *Fuca1*-deficient mice showed significant transcriptional upregulation (two- to fivefold) in the knockout mice for all analyzed genes involved in glycoprotein breakdown (Fig. S1G). Interestingly, in the kidney of knockout mice, transcript levels of the same genes remained more or less unaffected with the exception of β-hexosaminidase, α-mannosidase and α-L-fucosidase 2, all of which were significantly downregulated (Fig. S1H).

We further determined *in vitro* enzyme activities for β-hexosaminidase and α-mannosidase in various tissues derived from the same mice. α-mannosidase activity was markedly and significantly elevated in all tested organs from *Fuca1*-deficient mice (Fig. 1C), whereas β-hexosaminidase activity had a more tissue-specific pattern (Fig. 1D): in liver, β-hexosaminidase activity was comparable in both genotypes, whereas spleen, cerebrum and cerebellum from knockout mice showed a two- to threefold increase in β-hexosaminidase activity. Most remarkably, kidney β-hexosaminidase activity was twofold elevated in knockout mice, although its mRNA level was significantly downregulated, suggesting that the β-hexosaminidase enzyme was more stable in *Fuca1*-deficient mice.

Western blot analysis revealed that there was a twofold increase in the amount of the lysosomal hydrolase cathepsin D in cerebrum and kidney of *Fuca1*-deficient mice (Fig. 1E). Lamp1 expression was upregulated in cerebrum at the transcript and protein levels, as demonstrated by qPCR analyses (Fig. S1G) and western blot analyses (Fig. 1F). Interestingly, despite the total lack of α-L-fucosidase activity at neutral as well as acidic pH (Fig. 1B, Fig. S1D), the *Fuca2* transcript level was increased 3.3-fold in cerebrum (Fig. S1G). In contrast, the expression of the *Fuca2* transcript was reduced by 50% in the kidney of *Fuca1*-deficient mice (Fig. S1H). Under which conditions, if at all, this second fucosidase contributes to total fucosidase activity *in vivo* remains an open question.

### Macroscopic inspection of *Fuca1*-deficient mice

*Fuca1*-deficient mice were viable, fertile and born at nearly Mendelian inheritance of ∼23% (99 of 430 mice). Mice showed normal growth and weight gain, and were indistinguishable from wild-type mice until the age of ∼6 months. From this age onwards, *Fuca1*-deficient mice became progressively inactive, avoided any type of movement, became ataxic and developed a massive tremor. Macroscopic inspection of visceral organs of *Fuca1*-deficient mice revealed no obvious enlargement of visceral organs like liver, spleen, heart or kidney at the ages of 3.5 to 9 months. However, at 6 months of age, the urinary bladders of *Fuca1*-deficient mice, particularly of male mice, were enormously increased in size with an overall volume of 2-3 ml instead of 0.15-0.25 ml in the wild type, suggesting urological problems like urethral stricture or obstruction of the bladder neck because urinary creatinine concentrations of knockout mice (*n*=4) were comparable to wild-type (*n*=4) values ranging from 68 to 95 mg/dL. Owing to the general severe phenotype, and to avoid undue suffering, *Fuca1*-deficient mice were euthanized at 9-11 months of age at the latest.

### Urinary excretion and tissue storage of fucosylated glycoconjugates

**Fig. 2.**
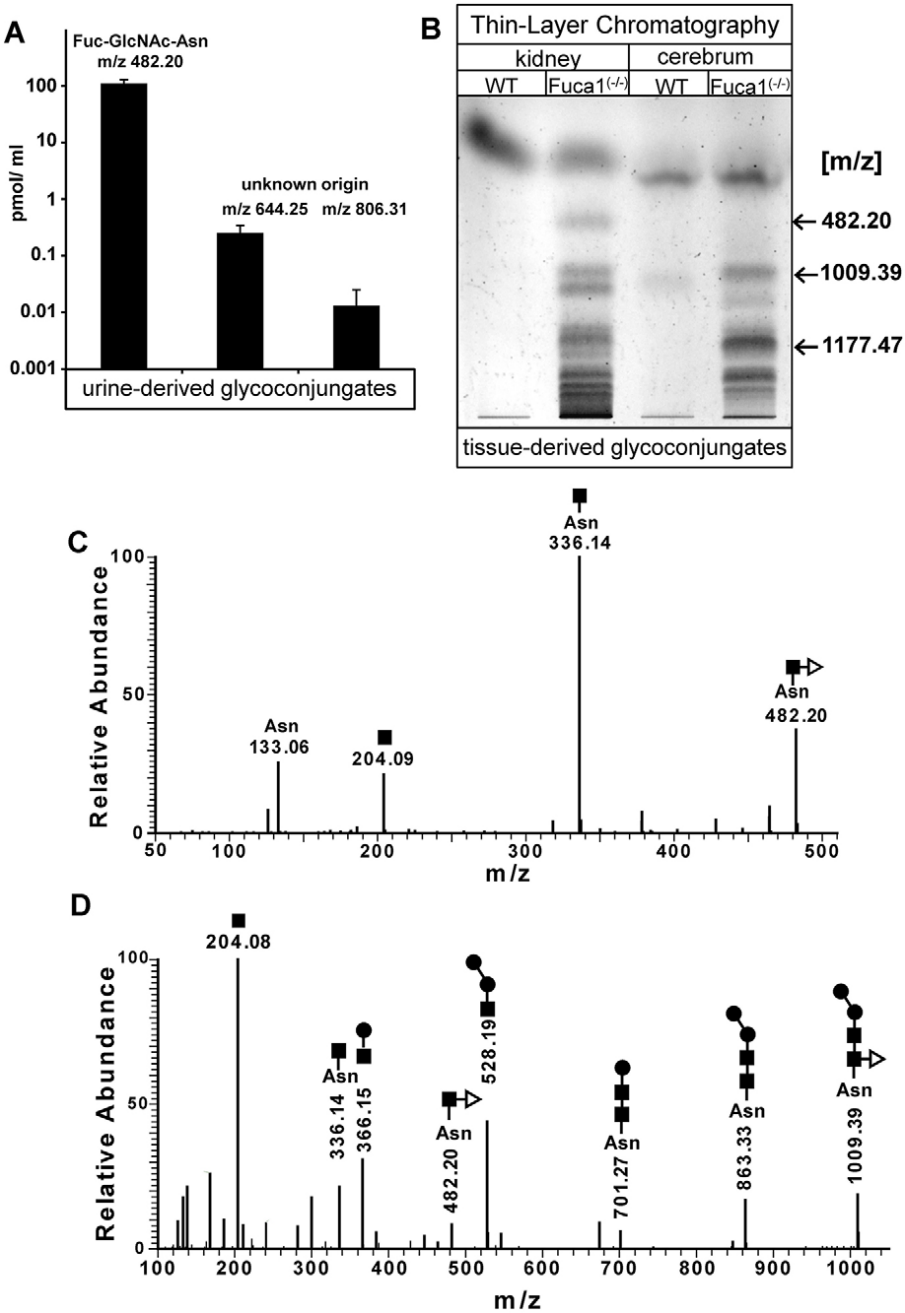
**Analysis of storage material.** (A) A spot urine test of *Fuca1*-deficient (−/−) mice at 9 months of age was performed and samples were directly analyzed with high-performance liquid chromatography mass spectrometry. *Fuca1*-deficient mice excretions contained fucosylated glycoasparagines, as well as other glycoconjugates of yet unknown origin. (B) Neutral oligosaccharides were isolated from kidney and cerebrum of 5-month-old mice and separated by using TLC. TLC-separated material was extracted from the plate and analyzed by liquid chromatography MS/MS. Two different fucosylated glycoasparagines were detected in *Fuca1^−/−^* tissues together with other glycocompounds of yet unknown origin. (C,D) MS/MS data of the TLC-separated glycoasparagines: Fuc-GlcNAc-Asn [*m*/*z* 482.20(M+H)^+^] and the pentasaccharide Man_2_-GlcNAc-(Fuc)-GlcNAc-Asn [*m*/*z* 1009.39(M+H)^+^] from *Fuca1^−/−^* tissues. The symbols for the monosaccharides of the detected glycans are as follows: N-acetylglucosamine (black square); mannose (black circle); fucose (open triangle); Asn, asparagine; WT, wild type.

A major biochemical hallmark of fucosidosis individuals, as a consequence of incomplete degradation of glycoproteins and glycolipids, is the accumulation of fucosylated glycoconjugates in tissues and their subsequent urinary excretion. Neutral oligosaccharides and glycoconjugates were extracted from mouse urine and tissues, respectively, and analyzed by performing mass spectrometry, either directly or after separation using thin layer chromatography (TLC). In the urine of knockout mice, but not of wild-type mice, the core-fucosylated glycoasparagine Fuc(α1,6)-GlcNAc(β1-N)-Asn [*m*/*z* 482.20 (M+H)^+^] was detected as the far-most abundant compound, whereas other structures [*m*/*z* 644.25 (M+H)^+^ and *m*/*z* 806.30 (M+H)^+^] of unknown origin appeared to much lesser amounts (Fig. 2A). Mass spectrometry analyses (MS/MS) of TLC-separated glycans from kidney and cerebrum of knockout mice identified Fuc-GlcNAc-Asn [*m*/*z* 482.20 (M+H)^+^] and, in addition, the asparaginyl-linked core fucosylated pentasaccharide Man_2_Fuc_1_GlcNAc_2_-Asn [*m*/*z* 1009.39 (M+H)^+^] (Fig. 2B-D). A further TLC-derived band with an *m*/*z* value of 1177.47, which would fit to a heptasaccharide composed of five hexose moieties, one HexNAc moiety and one fucose moiety, was exclusively found in knockout tissues, indicating a mixture of undigested fucosylated compounds.

### Histological analysis of visceral organs in *Fuca1*-deficient mice

**Fig. 3.**
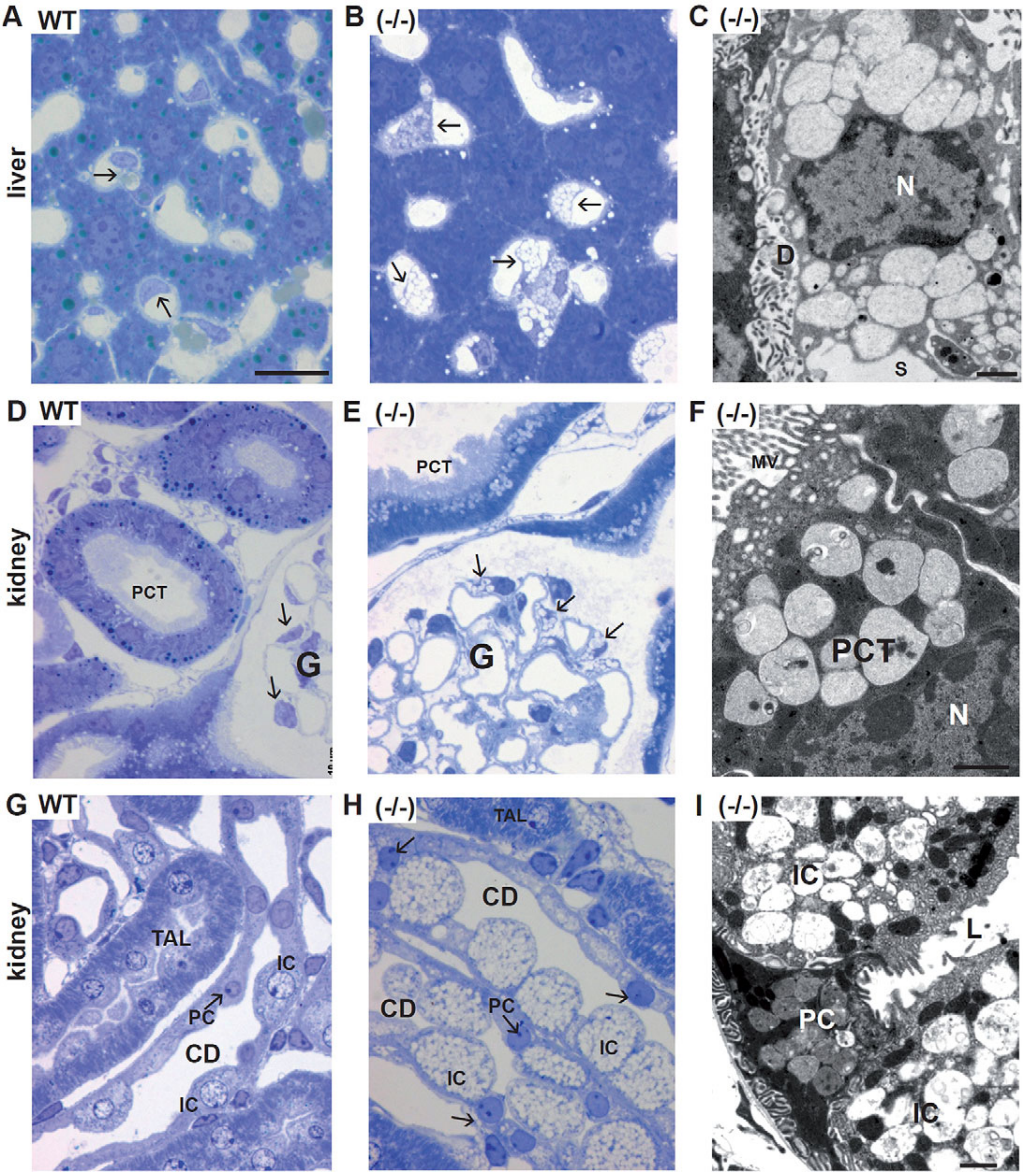
**Histopathology of liver and kidney in 5-month-old *Fuca1*-deficient mice.** Semi-thin sections stained with Toluidine Blue are shown in the left and middle columns. The panel column on the right-hand side (C,F,I) shows electron micrographs. (A-C) Liver, Kupffer cells (arrows). In *Fuca1*-deficient liver (−/−), Kupffer cells show numerous vacuoles, which are absent in the wild type. The hepatocytes appear inconspicuous upon light-microscopy analysis. The abnormal Kupffer cell vacuoles contain remnants of homogenous amorphous material of very low electron density. ‘D’, space of Disse; S, lumen of the sinusoid; N, nucleus. (D-F) Renal cortex. In *Fuca1*-deficient mice (−/−), podocytes (arrows) of the glomerulus (‘G’) and proximal convoluted tubules (PCT) display cytoplasmic vacuoles, the latter contain amorphous material of moderate electron density. MV, microvilli; N, nucleus. (G-I) Renal outer medulla. Thick ascending limbs (TAL) and collecting ducts (CD) are shown. The CD epithelium is composed of principal cells (PC, arrows) and intercalated cells (IC). In the *Fuca1*-deficient mouse the IC are severely vacuolated, whereas the PC appear inconspicuous at LM level. At the electron microscopy level, the PCs also show vacuolization (I) in which the accumulated material resembles that in PCT. The vacuoles in the IC are almost empty. In the TAL epithelium, abnormal vacuoles do not occur. L, lumen of the collecting duct. WT, wild type. Scale bars: 20 µm (all light micrographs); 1 µm (electron micrographs).

We analyzed various tissues of *Fuca1*-deficient mice and wild-type mice at 5 to 9 months of age with light-microscopy analysis and transmission electron microscopy. Mainly, translucent cytoplasmic storage vacuoles indicated that water-soluble storage material is the predominant substrate accumulating in *Fuca1*-deficient mice. In most tissues, these empty vacuoles were easily detectable in semi-thin sections with light-microscopy analysis. In the liver, Kupffer cells and sinusoidal endothelial cells showed large empty vacuoles (Fig. 3A-C). The hepatocytes, which are heavily affected in human and mouse α-mannosidosis (Monus et al., 1977; Stinchi et al., 1999) and in α-glucosidase deficiency (Pompe disease) (Bijvoet et al., 1998; Hers, 1963), appeared normal upon light-microscopy analysis, although low storage was observed under electron microscopy (Fig. S2C). In the kidney of *Fuca1*-deficient mice, glomerular podocytes, glomerular mesangium cells and convoluted proximal tubules exhibited moderate lysosomal storage pathology (Fig. 3E,F), whereas the intercalated cells of the collecting ducts were severely vacuolated (Fig. 3H,I). The urinary bladder showed large empty vacuoles, particularly in the superficial umbrella cells of the urothelium (Fig. S2A). In the spleen, sinusoidal endothelial cells, trabecular fibroblasts and lymphocytes were affected (Fig. S2B). Moreover, storage vacuoles were prominent in the epithelial cells of many other organs, such as in the pancreas and gall bladder (Fig. S2D). In a few cell types – such as hepatocytes, principal cells of renal collecting ducts as well as cells of the convoluted proximal tubules – the storage vacuoles contained very homogenous amorphous material of moderate electron density (Fig. 3I), thus differing from the empty vacuoles seen in the majority of affected cells.

In conclusion, pathological storage was observed in many visceral organs of *Fuca1*-deficient mice, but number, size and content of storage vacuoles differed in a cell-type-specific manner. Remarkably, a few tissues and cell types were largely exempt from aberrations in storage, such as bone cells, renal thick ascending limbs of Henle's loop, skeletal muscle and heart muscle.

### Neuropathology

**Fig. 4.**
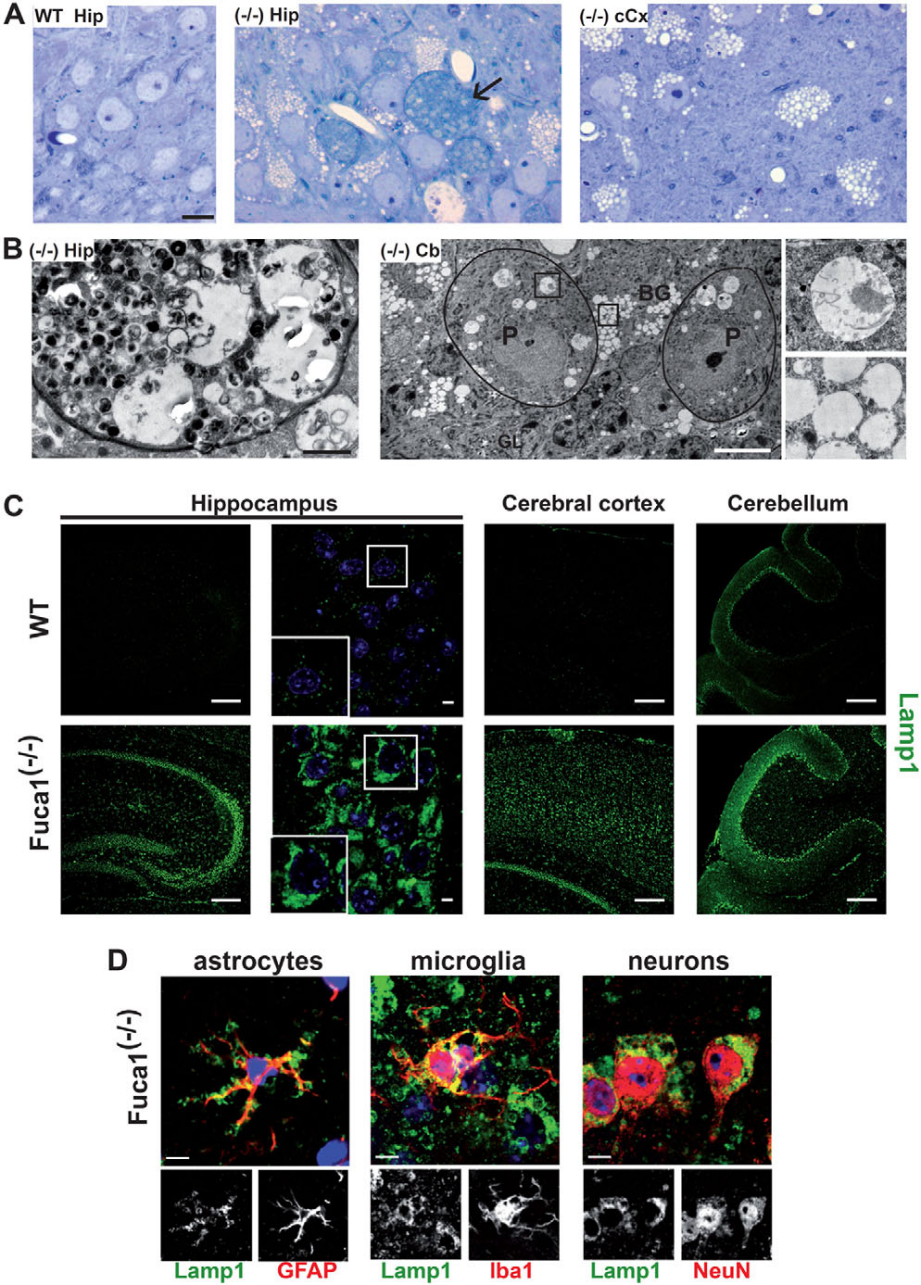
**Neuropathology in the central nervous system of *Fuca1*-deficient mice.** (A) Storage vacuoles of 9-month-old *Fuca1*-deficient (−/−) mice (light microscopy, semi-thin sections, Toluidine Blue); wild-type hippocampus WT Hip (CA3 region): the perikarya contain some dense bodies, and cytoplasmic vacuoles are absent. Hippocampus of *Fuca1*-deficient mouse (−/−)Hip (CA3 region): the perikarya contain numerous empty cytoplasmic vacuoles. Arrow points to one of the three axon spheroids present in this picture. (−/−)cCX, cerebral isocortex of the same mouse: the neuronal perikarya show empty vacuoles. Scale bars: 10 µm. (B) Ultrastructural analysis of storage material. (−/−)Hip, hippocampal axon spheroid: the axoplasm contains polymorphous material. The same mouse as shown in A was used. (−/−)Cb, low electron microscopy magnification of cerebellar cortex at the age of 3.5 months: Purkinje cells (P, encircled) show relatively large vacuoles with some floccular storage material (upper inset). The Bergmann glia cells (BG) are filled with small empty vacuoles (lower inset). GL, granular layer. Scale bars: 1 µm. (C) Immunofluorescence staining of brain sections showed an increased amount of lysosome-associated membrane protein 1 (Lamp1) in the hippocampus, cerebral cortex and cerebellum of 3.5-month-old *Fuca1^−/−^* mice. Scale bars: 200 µm (overview images); 5 µm (magnified images of the hippocampus on right). Nuclei were stained with DAPI (blue). (D) Immunofluorescence staining of Lamp1-positive cells with different neural-cell markers (GFAP for astrocytes, Iba1 for microglia, NeuN for neurons) revealed that all neural cells types were affected by storage pathology. Scale bars: 5 µm.

Neuropathological and ultrastructural studies of human fucosidosis are rare, and they mainly describe vacuolation with electron-lucent to moderate granular content of cerebral and cerebellar neurons and glial cells. In addition, loss of neuron populations in the thalamus, dentate nucleus and particularly Purkinje cells of the cerebellum has been described (Bugiani and Borrone, 1976; Durand et al., 1969; Loeb et al., 1969). We examined the cerebral cortex, hippocampus, cerebellar cortex, spinal cord and selected regions of white matter of our mouse model by using light-microscopy analysis and, in selected cases, also by electron microscopy. In the *Fuca1*-deficient mice, storage vacuoles were most prominent in neuronal perikarya (Fig. 4A,B), astrocytes, amoeboid microglia and in the ependymium, as well as in the choroid plexus epithelium. Another observation, which has been reported also for other animal models of LSDs, such as α-mannosidase-deficiency and mucopolysaccharidoses (Stinchi et al., 1999; Wilkinson et al., 2012), was the occurrence of axon spheroids – i.e. local axonal swellings. The axoplasm in the spheroids was crowded with disoriented neurotubules, neurofilaments and heterogeneous material (Fig. 4B). Axon spheroids were encountered both in gray matter – e.g. in the hippocampal CA3 region – as well as among the perikarya of the nucleus gracilis and in the white matter – e.g. corpus callosum.

Immunostaining for the lysosomal marker protein Lamp1 revealed a massive increase in Lamp1-positive structures in *Fuca1*-deficient mice, indicating an extended endosomal-lysosomal network (Fig. 4C). A detailed analysis of heavily Lamp1-positive cells by immunostaining using cell-specific markers identified astrocytes (glial fibrillary acidic protein, GFAP), microglia (Iba1) and neurons (NeuN) as affected cell types (Fig. 4D).

**Fig. 5.**
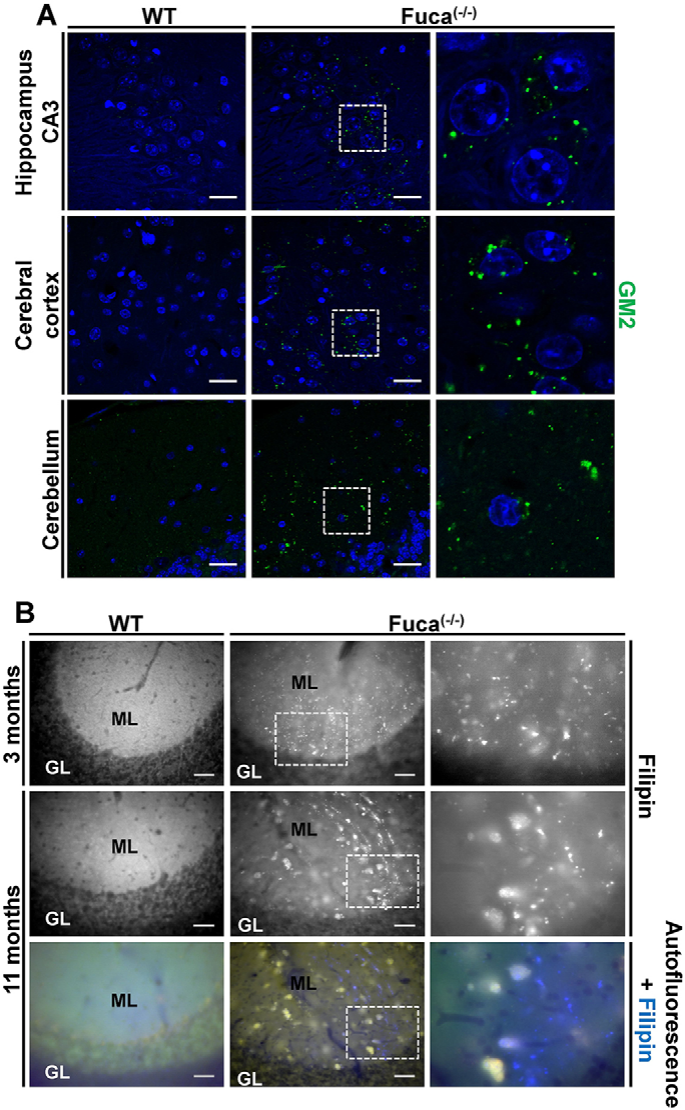
**Secondary storage of GM2 ganglioside and cholesterol in Fuca1-deficient mice.** (A) GM2 immunoreactivity is prominent in the CNS of 3-month-old *Fuca1*-deficient (−/−) mice but not in wild-type mice (WT). Magnified images of the boxed areas (∼2.5×) are shown on the right. Scale bars: 25 µm. (B) Accumulation of free cholesterol was shown by filipin staining, particularly in the molecular layer (ML) but not in the granular layer (GL) of the cerebellum of knockout mice at the age of 3 and 11 months. By contrast, WT mice were devoid of such large cholesterol deposits. Magnified images of the boxed areas (∼2.5×) are shown on the right. Scale bars: 50 µm.

Secondary storage of lipids is often part of the neuropathologic cascade in mouse models for LSDs, as documented for mucopolysaccharidoses and α-mannosidosis (Damme et al., 2011; Walkley, 2004). In 3-month-old mice, immunoreactivity for the GM2 ganglioside was already prominent in vesicular structures of the CA3 region of the hippocampus, the cerebral cortex and in the molecular layer of the cerebellum of knockout mice, whereas no such structures were detected in wild-type mice (Fig. 5A). By using the cholesterol-binding fluorescent dye filipin, we proved the accumulation of free cholesterol in the molecular layer of the cerebellum in 3-month-old and 11-month-old *Fuca1*-deficient mice but not in wild-type mice (Fig. 5B).

Analyses of unstained sections of 11-month-old mice revealed autofluorescent storage material in cerebrum and cerebellum of knockout mice, with the cerebellar staining primarily appearing in cellular structures of the molecular layer (Fig. S3A). Histochemical Sudan Black B specific staining for lipofuscin – i.e. aggregates of oxidized proteins, lipids and metal ions – was positive also in comparable cellular structures in the molecular layer of the cerebellum (Fig. S3B).

De- or hypomyelination has been described for some severely affected individuals and in the dog model, respectively. Of note, such myelination defects have not been detected in the CNS of *Fuca1*-deficient mice (Fig. S4).

**Fig. 6.**
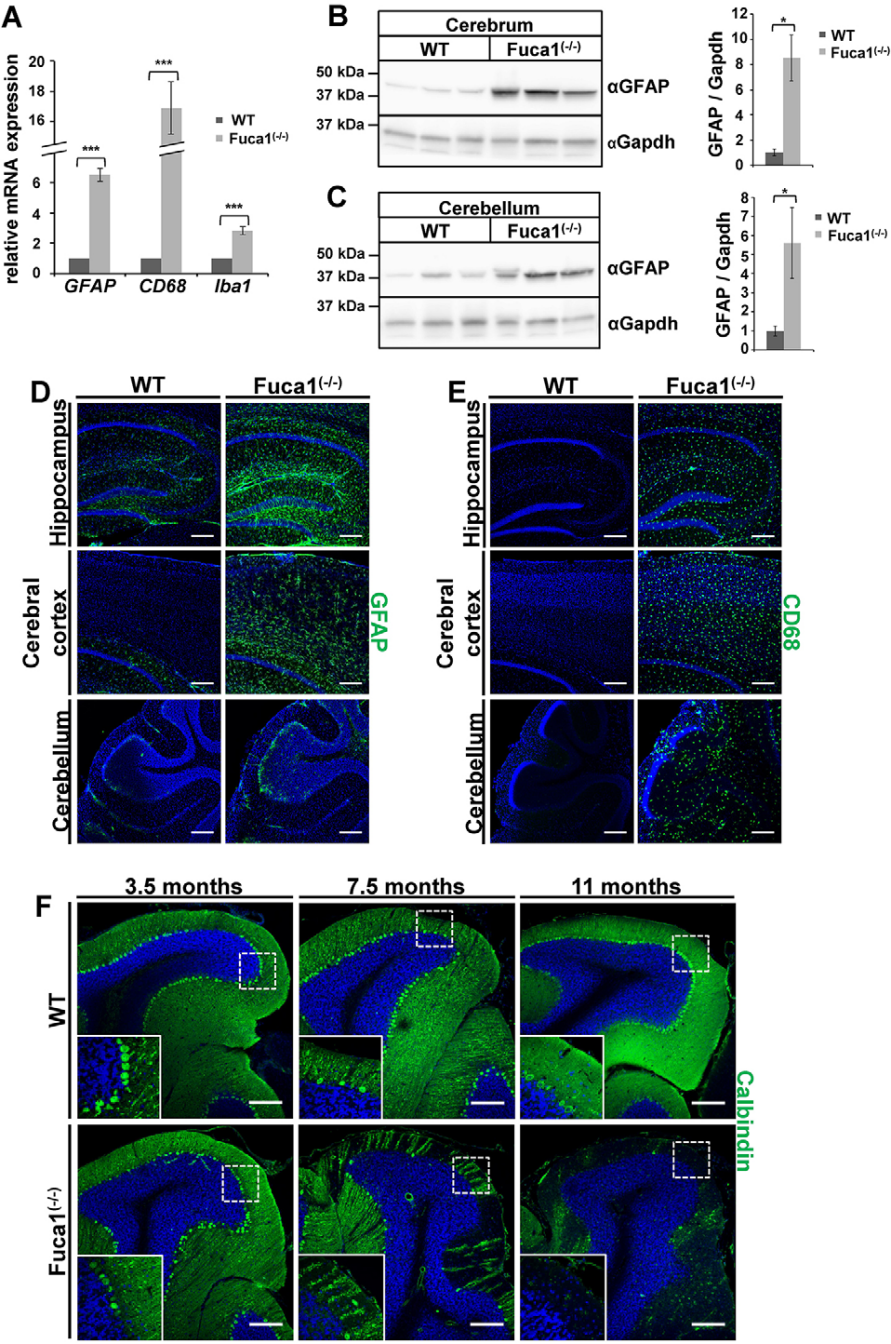
***Fuca1*-deficient mice exhibit neuroinflammation and loss of Purkinje cells.** (A) qPCR analysis of whole brain cDNA showed elevated mRNA levels for *Gfap*, *Cd68* and *Iba1* in 5-month-old *Fuca1*-deficient (−/−) mice. *n*=3 technical replicates. (B,C) Increased amounts of GFAP protein were found in *Fuca1^−/−^* cerebrum and cerebellum at 5 months of age, as shown by immunoblotting. GAPDH was used for normalization. *n*=3 biological replicates. (D,E) Immunofluorescence staining revealed increased levels of GFAP (D) and CD68 (E) in the hippocampus, cerebral cortex and cerebellum of *Fuca1^−/−^* mice at 3.5 months of age. (F) Section of wild-type and *Fuca1^−/−^* cerebellum at 3.5, 7.5 and 11 months of age revealed a massive decrease for the Purkinje cell marker calbindin, indicating a progressive loss of Purkinje cells in *Fuca1^−/−^* mice. The nuclei were stained with DAPI. Scale bars: 200 µm. Data are mean±s.d. *P<0.05, ***P<0.0005 (two-tailed *t*-test).

Lysosomal storage CNS pathology is often characterized by inflammation, as indicated by the activation of microglia and astrocytes. In knockout mice, gene expression of the astrocytic marker GFAP, the microglia and macrophage marker *Cd68* (macrosialin) and of the microglia- and macrophage-specific Ca^2+^-binding protein *Iba1* were significantly increased as determined by qPCR analyses of total brain (Fig. 6A); GFAP expression was also increased at the protein level, as detected by western blotting (Fig. 6B,C). Immunofluorescent analyses clearly confirmed an increase in GFAP- and also CD68-positive cells in the cerebral cortex and the hippocampus, as well as in the cerebellum (Fig. 6D,E). CD68 was detected throughout the entire cerebellum, whereas GFAP staining was most prominent in Bergmann-glia cells, which are located along the border of molecular layer and granular layer (Fig. 6D, bottom panel). In several animal models of LSDs, a progressive loss of Purkinje cells is accompanied by reactive and progressive astrogliosis in affected areas (D'Hooge et al., 1999; Damme et al., 2011; Kollmann et al., 2012). In order to evaluate the progressive loss of Purkinje cells, cerebellar sections from mice of different ages were stained for the Purkinje cell marker calbindin. Indeed, an age-dependent decline of Purkinje cells from 3.5 months to 7.5 months of age was observed, leading to a nearly complete loss of Purkinje cells at 11 months of age (Fig. 6F).

### Behavioral studies

**Fig. 7.**
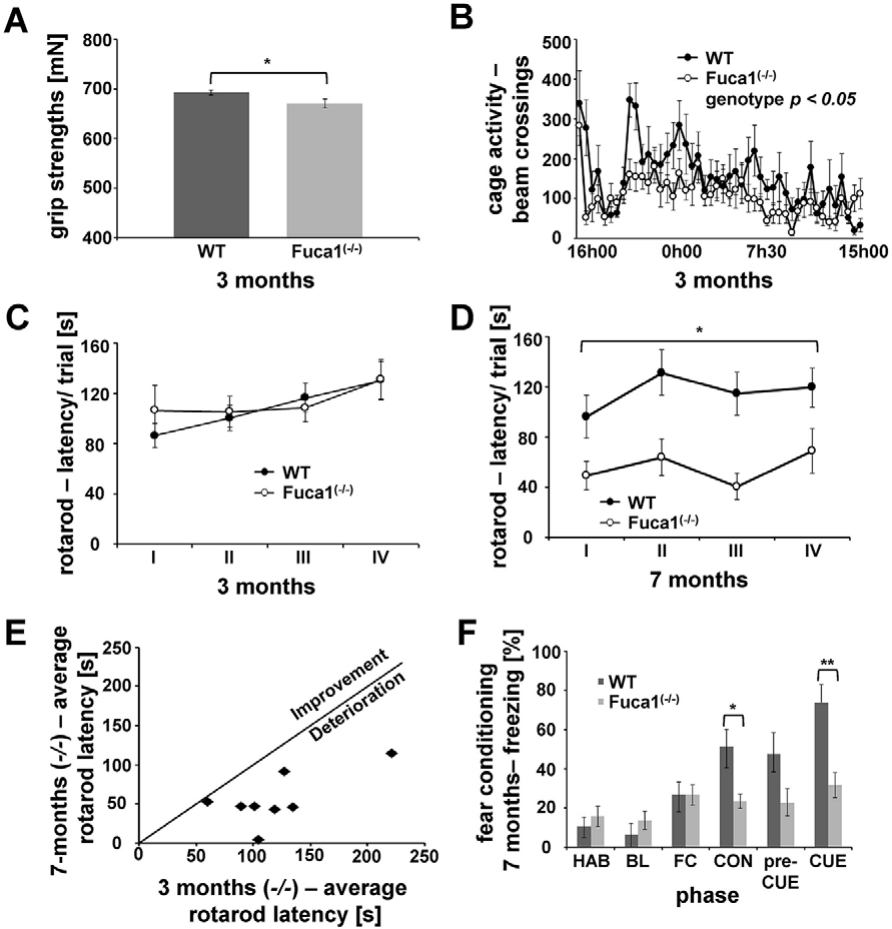
***Fuca1*-deficient** **mice show behavioral deficits.** (A) 3-month-old *Fuca1*-deficient (−/−) mice showed reduced forelimb strength. (B) Home cage activity was recorded during a 23-h session, indicating general hypoactivity in 3-month-old *Fuca1^−/−^* mice. (C) A rotarod test was performed with 3-month-old mice and showed no difference in drop latency between the genotypes during four (I-IV) consecutive trials. (D) The rotarod test with 7-month-old animals showed a decreased average drop latency across four consecutive trials for the *Fuca1^−/−^* mice. (E) Scatter plot reflecting rotarod drop latency of *Fuca1^−/−^* mice at 3 and 7 months of age, indicating progressive impairment of motor function in all investigated animals. (F) No difference in baseline freezing levels or fear acquisition was observed in *Fuca1*-deficient (−/−) mice. However, freezing percentage decreased during fear memory evaluations in *Fuca1*-deficient (−/−) mice, reflecting both impaired contextual and cued fear memory. HAB, habituation; BL, baseline; FC, fear conditioning; CON, context fear; preCUE, pre-cued fear; CUE, cued fear; mean±s.e.m.; *n*=15 for 3-month-old mice; *n*=10 (WT) and *n*=8 (*Fuca1^−/−^*) for 7-month-old mice. Only significant effects with regard to genotype are highlighted. Asterisks indicate significant difference between Fuca1-deficient mice and wild-type controls, *P<0.05; **P<0.01 (two-tailed *t*-test for A,F; repeated-measures ANOVA for B-E).

Because neuropathological alterations are often reflected by behavioral deficits, *Fuca1*-deficient mice were analyzed using a series of behavioral tests. A grip test was included as an index of neuromuscular function and fine motor skills. At the age of 3 months, *Fuca1*-deficient mice showed reduced forelimb grip strength (Fig. 7A; *t*-value=2.12, *P*<0.05, two-tailed *t*-test). Furthermore, recurrent paw misplacement was observed in 40% of knockout mice (6 out of 15) in comparison to 7% of wild-type animals (1 out of 15). Analysis at the age of 7 months showed a similar deficit in grip strength, without detectable progression [Fig. S5A; *t*-value=2.93, *P*<0.05, two-tailed *t*-test; paw misplacements, 50% for knockout mice (4 out of 8), 0% for wild-type mice (0 out of 10)]. Analysis of home-cage activity in 3-month-old animals revealed a significant effect of the time of day (Fig. 7B; *F*-value=4.28, *P*<0.001, repeated-measures ANOVA), reflecting an initial exploratory peak and a characteristic pattern of circadian activity. However, knockout mice showed a hypoactive phenotype (Fig. 7B; *F*-value=4.33, *P*<0.05, repeated-measures ANOVA) because typical movement peaks were less pronounced or absent. No obvious progression was observed when mice were tested at the age of 7 months (Fig. S5B).

Motor performance and balance were evaluated in the rotarod test. At the age of 3 months there was a significant effect of trial (Fig. 7C; *F*-value=5.53, *P*<0.01, repeated-measures ANOVA), i.e. drop latencies increased during subsequent trials, reflecting considerable motor learning in both genotypes. A lack of main and interaction effects related to genotype were revealed by similar rotarod performances of knockout and wild-type mice. Evaluation of 7-month-old animals again revealed a significant effect of trial (Fig. 7D; *F*-value=4.64, *P*<0.01, repeated-measures ANOVA). Mice generally performed better during trials II and IV. Importantly, *Fuca1*-deficient mice at this age showed significantly decreased drop latencies in comparison to wild-type controls (*F*-value=8.50, *P*<0.05, repeated-measures ANOVA). Longitudinal analysis of knockout mice indeed showed progressive impairment between 3 months and 7 months of age (*t*-value=5.20, *P*<0.01, two-tailed *t*-test). Individual scores are plotted in Fig. 6E to illustrate that this decline was evident in all investigated *Fuca1*-deficient animals.

Cognitive ability was evaluated by using a contextual and cued fear conditioning protocol (Fig. 7F). Both genotypes showed similar freezing behavior during the preparatory phases of the experiment (habituation and baseline). The number of times that mice froze (as a percentage) increased independent of genotype during fear conditioning (*F*-value=9.37, *P*<0.01, repeated-measures ANOVA) reflecting successful fear acquisition. During the last day of testing, mice were first placed in the apparatus under similar circumstances to the habituation phase (no stimulus presentations) to evaluate freezing behavior in the original context. *Fuca1*-deficient mice showed decreased freezing behavior during this context phase, indicating impaired contextual fear memory (*t*-value=2.96, *P*<0.05, two-tailed *t*-test). Different environmental alterations were made for the pre-cue phase, but freezing behavior was very similar to that in the contextual phase for both genotypes. Despite this generalization of fear in response to the newly created context during the pre-cue phase, presentation of the auditory stimulus (the cue) elicited a substantial increase of freezing behavior in wild-type mice during the cue phase. However, this reflection of cued fear memory proved very weak or absent in knockout mice as compared to wild-type controls (*t*-value=3.62, *P*<0.01, two-tailed *t*-test).

## DISCUSSION

Lysosomal α-L-fucosidase is the key enzyme in the degradation of fucosylated glycoconjugates, and its deficiency results in the neurodegenerative LSD fucosidosis in human as well as in dogs and domestic breeds of cat. We generated a *Fuca1*-deficient mouse model and investigated *Fuca1* deficiency at the genomic, transcript and enzyme activity levels. The clinical course of our fucosidosis mouse model resembled the milder type-2 fucosidosis variant in humans and was particularly characterized by defects of the CNS, leading to motor and mental impairments. Additional features of human fucosidosis, such as dysostosis multiplex, coarse features, ocular abnormalities and hearing loss, were not present in the mouse model, or they need to be revisited in more detail in the future (for comparison of pathological features between human and mouse see also Table S1).

At the biochemical level, the total lack of α-L-fucosidase activity resulted in the lysosomal accumulation of fucosylated glycoasparagine molecules in various knockout tissues and in the excretion of fucosylated glycoconjugates in urine. As in fucosidosis-affected individuals, the α1,6-fucosylated glycoasparagine Fuc-GlcNAc-Asn represented, by far, the most abundant urine-excreted glycoconjugate in fucosidosis mice, which is not found in healthy humans and control mice (Michalski et al., 1991; Strecker et al., 1978, 1977; Tsay and Dawson, 1976; Tsay et al., 1976). It is known that α1,6-(core)-fucosylation sterically inhibits the lysosomal aspartyl-glucosaminidase (AGA; EC 3.5.1.26). Thus, fucosylated glycoasparagine molecules resist lysosomal N-glycan degradation and become detectable in human fucosidosis or related glycoproteinoses (Haeuw et al., 1994; Noronkoski and Mononen, 1997). We also identified some low-abundance excreted compounds of unknown nature in the urine of fucosidosis mice with masses (*m*/*z*) of 644.25 and 806.31, which do not match with masses of N-linked oligosaccharides of any commonly known structure. In future, liquid chromatography MS/MS analysis should help to unravel the nature of these compounds as fucosylated oligosaccharides that might be derived from O-linked glycans or from glycolipids.

In humans, more than 20 different glycoasparagine species, but also various oligosaccharides, have been shown to be stored in tissues and/or to be excreted in the urine of fucosidosis individuals (Michalski and Klein, 1999; Nishigaki et al., 1978; Yamashita et al., 1979). So far, we have not detected oligosaccharides with terminal α1,2-, α1,3- or α1,4-linked fucose residues, which might reflect a lower degree of terminal fucosylation in mouse tissues. Several studies of storage material from human brain and urine have also identified glycolipids, such as gangliosides, as well as Lewis^X^ antigens (Dawson and Spranger, 1971; Kin, 1987; Tsay and Dawson, 1976; Tsay et al., 1976). With regard to lipids, the accumulation of the non-fucosylated GM2 ganglioside as well as the cholesterol deposits in our fucosidosis mouse model are due to secondary storage, which is a common feature of numerous lysosomal diseases (Walkley and Vanier, 2009) and might interfere with normal regulatory circuits that are controlled by the endosomal-lysosomal system, such as Ca^2+^ homeostasis, autophagy, and lipid synthesis and trafficking (Ballabio and Gieselmann, 2009; Vitner et al., 2010). Besides the storage of lipids, we also observed autofluorescent material, which, most likely, represents lipofuscin-like ceroids, as reported for many neuronal ceroid lipofuscinoses, mucopolysaccharidoses and the glycoproteinosis α-mannosidosis (Damme et al., 2011; Jalanko and Braulke, 2009; Martins et al., 2015). Unfortunately, neither human brain biopsies nor the brain of the dog model have been inspected so far for secondary lipid storage or the presence of lipofuscin-like ceroids.

As expected from the human disease and the dog model, the CNS of the fucosidosis mouse model was severely affected in terms of a massive presence of storage vacuoles, enlargement of the endosomal-lysosomal system, signs of inflammation and a progressive loss of Purkinje cells that in combination with astrogliosis contributes to significant psychomotor and memory deficits. Indeed, fucosidosis mice were hypoactive and showed reduced grip strength at 3 months of age. Gross neuromotor performance in the rotarod test was still intact at that time, but became clearly impaired at 7 months, coinciding with the progressive decrease of cerebellar Purkinje cells. In addition, the described hippocampal pathology is likely to contribute to the deficits observed in contextual and cued fear memory, which clearly indicate cognitive dysfunction. Therefore, the murine phenotype accurately reflects progressive motor impairment and intellectual disability, the behavioral hallmarks of human fucosidosis.

Interestingly, severely affected fucosidosis individuals (Galluzzi et al., 2001) and the dog model exhibit myelin deficits due to the loss of oligodendrocytes in the CNS, including the cerebrum, the corpus callosum and particularly the cerebellum (Fletcher et al., 2014), which can not be seen in our mouse model. Such a species-specific difference in myelination pathology has also been described for the lysosomal storage disorder metachromatic leukodystrophy (MLD, arylsulfatase-A-deficiency), where the MLD mouse model exhibits storage pathology and histological alterations in many parts of the CNS, including oligodendrocytes, but lacks prominent demyelination in the CNS (Gieselmann et al., 1998; Hess et al., 1996).

The heterogeneity of α-L-fucosidase substrates is not only reflected by the different fucosylated compounds, which can be detected as storage material in tissues or in the urinary excretions, but also by the remarkable cell-specific variability in the content of the storage vacuoles. Their appearance varied from empty (or translucent) vacuoles to vacuoles with electron-dense granular or even lamellar material, as is observed in human, dog and also mouse fucosidosis (Hartley et al., 1982; Willems et al., 1991). However, the majority of affected cell types in visceral organs and in the CNS of the fucosidosis model presented with empty vacuoles, which are ascribed to water-soluble storage material such as glycoasparagines or oligosaccharides. However, the extent of vacuolation also varied remarkably with massive storage pathology in Kupffer cells and in the sinusoidal endothelium of the liver and the urothelium, as well as in several cell types in the kidney, whereas other cell types exhibited only little or no vacuolation at all.

Despite the total lack of α-L-fucosidase activity and the substantial neuropathology, our mouse model displays the milder form of the disease (type 2). An obvious clinical phenotype was not observed before 6 months of age, whereas in humans, the early onset type-1 fucosidosis is characterized by a rapid progression of neurological symptoms and death within the first decade of life (Kousseff et al., 1976; Willems et al., 1991). Furthermore, in humans, the severity of the disease, apparently, neither correlates with residual α-L-fucosidase activity nor with genetic heterogeneity (Willems et al., 1991), suggesting that other factors or modifiers have a strong impact on the severity of the clinical course. Of note, several other knockout mouse models for LSDs such as α-mannosidosis, arylsulfatase B-deficiency (MPS VI) or metachromatic leukodystrophy also develop clinical features that resemble the milder phenotype in human rather than the severe form (Evers et al., 1996; Hess et al., 1996; Stinchi et al., 1999).

One putative relevant modifier for fucosidosis could be α-L-fucosidase 2, which might represent a second lysosomal fucosidase, as described recently upon analysis of the lysosomal proteome (Lübke et al., 2009) and as further characterized with localization studies using mCherry-fusion constructs (Huang et al., 2014). In spite of the significant upregulation of *Fuca2* at the transcript level, at least in cerebrum, no fucosidase activity at all was measurable in the *Fuca1* knockout, whatever tissue or pH condition we tested. This clearly argues against a role of *Fuca2* as a modifier of disease severity.

Because only very few data are available from human fucosidosis individuals, the dog model has already been very valuable. Analysis of the dog model has furthered understanding of the biochemistry, pathology and neurological alterations underlying fucosidosis (Abraham et al., 1984; Fletcher et al., 2014; Fletcher and Taylor, 2016; Kondagari et al., 2011b), as well as the development of therapeutic approaches such as bone marrow transplantation and intracisternal ERT, overcoming the blood-brain barrier (Kondagari et al., 2011a; Taylor et al., 1992). Nevertheless, our mouse model, which also reflects many aspects of human fucosidosis, has several advantages over the dog model, such as easier breeding, a defined genetic background, a shorter life span and much lower body weight, which in total are cost-reducing factors, in particular for the establishment of ERT. Production of a sufficient amount of recombinant enzyme for high-dose and long-term treatments in mice is much more economic and feasible than in the dog model. Mouse models are widely accepted for establishing ERT approaches for LSDs, including high-dose intravenous and chronic ERT, as well as intraventricular or intrathecal ERT for those LSDs that present with neurological symptoms. In order to initiate any kind of therapeutic approaches in human, and to set up a valid natural history study, a global data base of fucosidosis-affected individuals has to be coordinated and established very soon.

## MATERIALS AND METHODS

### Generation of *Fuca1*-deficient mice

*Fuca1*-deficient (*Fuca*^−/−^) mice were generated by using a gene-targeting strategy, as shown in Fig. S1A. A 6-kb fragment of the murine *Fuca1* gene including exon 1 and exon 2 was amplified from genomic DNA using the primer pair E1fw and E1rev (Table S1), and was cloned into the pKO Scrambler V901-DT vector (Agilent Technologies, Santa Clara, CA, USA). *Afe*I restriction sites were used to insert a neomycin-resistance cassette (neo). The knockout construct was electroporated into murine embryonic stem cells [D3 (129S2/SvPas)] (Doetschman et al., 1985), and resulting geneticin (G418)-resistant embryonic stem cell clones were screened for homologous recombination by performing PCR. The 5′ homologous recombination site was validated using the primer pair F4 and AK31as (Table S2), whereas the 3′ homologous recombination site was identified using a nested PCR approach with the primer pairs F1 and R2, and AK31 and R1 (Table S2). Embryonic stem cells were microinjected into blastocysts (strain C57BL/6) and implanted into pseudo-pregnant mice. The resulting chimeras were crossed with C57BL6 mice. Germline transmission was validated, and the heterozygous offspring were intercrossed to obtain homozygous mice with a mixed genetic background (C57BL/6 and 129S2/SvPas).

The gene knockout was validated on genomic DNA by using a nested PCR approach and subsequent sequencing of the PCR products on the 5′ homologous recombination site (primer pairs F4 and R3, and F4 and Ak30, Table S2). Routine genotyping was performed using the primers F2, AK30-R and R3 (Table S2) in a multiplex PCR reaction.

The mice were housed under standard conditions in a pathogen-free animal facility at Bielefeld University. The experiments were performed with mice in a mixed genetic background using littermates as controls. All procedures and experiments in mice were performed in accordance with local guidelines and were approved by local authorities.

### cDNA synthesis and qPCR

For mRNA isolation the RNeasy Midi Kit (Qiagen, Hilden, Germany) was used, and cDNA was subsequently synthesized using the iScript Kit from BioRad (Hercules, CA, USA). The PCR was performed in a Step One Plus Real-Time PCR cycler instrument (Thermo Fisher Scientific, Waltham, MA, USA) using the Kapa Sybr Fast Universal Kit (PeqLab, VWR, Erlangen, Germany). All primer pairs are listed in the Table S2.

### Tissue homogenates

Mouse tissue (150 mg) was homogenized in a 20-fold volume of TBS containing 0.5% Triton X-100 (v/v) as well as protease inhibitors by performing five strokes with a Teflon pestle using a Potter S homogenizer (Braun, Melsungen, Germany) followed by subsequent sonification at 4°C (three 20-s pulses, 40% intensity; Sonifier 450, Branson Ultrasonics, Danbury, CT, USA). After incubation on ice for 30 min, the homogenates were centrifuged at 18,000 ***g*** for 15 min at 4°C. The supernatant was used for activity assays or immunoblotting. Protein concentration was determined using the DC Protein Assay (Bio-Rad).

### Isolation of lysosome-enriched fractions

Lysosomal fractions containing intact tritosomes were isolated as described previously (Wattiaux et al., 1963). 0.5% Triton X-100 (v/v) was added, and the tritosomes were lysed with sonification at 4°C (three 20-s pulses, 40% intensity; sonifier 450, Branson Ultrasonics).

Lysosomal fractions from brain were isolated as described elsewhere (Caimi et al., 1989). Briefly, a whole mouse brain was homogenized in 4 ml SED buffer (0.32 M sucrose containing 1 mM EDTA) in a Potter S homogenizer (Braun; Melsungen) by performing five strokes with a Teflon pestle rotating at 500 rpm. The homogenate was cleared by centrifugation at 1000 ***g*** for 10 min. The pellet was washed with 3 ml SED buffer and again centrifuged. Both supernatants were combined, and intact organelles were pelleted at 17,500 ***g*** for 1 h. The pellet was resuspended in 1 ml SED buffer. Percoll was made isocratic by adding 9 ml Percoll (Amersham Bioscience, Uppsala, Sweden) to 1 ml 2.5 M sucrose containing 10 mM EDTA (100% Percoll) and diluted to 27% (v/v) with SED buffer. The resuspended organelles (1 ml) were layered over 9 ml of 27% Percoll solution and centrifuged for 90 min at 20,000 ***g***. The gradient was collected from top to bottom in eight fractions of 1-ml volumes and four fractions of 0.5-ml volumes. 0.1% Triton X-100 (v/v) was added, and the organelles were lysed by sonification as mentioned above. The lysosome-enriched fraction was identified by determining the specific β-hexosaminidase activity. All work was performed at 4°C.

### α-L-fucosidase activity assay

α-L-fucosidase activity was measured using the artificial pseudosubstrate 4-methylumbelliferyl-α-L-fucopyranoside (4-MU-Fuc; Carbosynth, Compton, UK). Tissue homogenates (10 µl) or lysosome-enriched fractions (5 µl for liver or 25 µl for brain) were incubated with 150 µl of 0.75 mM 4-MU-Fuc in 0.1 M sodium citrate pH 5.5 including 0.2% BSA and 0.04% sodium azide for 16 h (tissue homogenates) or 6 h (liver lysosomes) or 4 h (brain lysosomes) at 37°C and stopped by the addition of 150 µl of 1 M sodium carbonate (pH 10.4). The amount of liberated 4-MU was determined by fluorescence measurements (excitation: 360 nm; emission: 465 nm) using the Infinite 200 microplate reader (Tecan, Männedorf, Switzerland) and calculated using a standard curve (0–10 nmol of 4-MU). If necessary, tissue homogenates were diluted between 1:10 and 1:100 to reach a linear range. For analysis of α-L-fucosidase pH dependency, McIlvaine's buffer was used instead of 0.1 M sodium citrate and adjusted to the stated pH values.

### α-mannosidase and β-hexosaminidase activity assay

The activity of α-mannosidase and β-hexosaminidase was determined by measuring the amount of liberated p-nitrophenol from the enzyme-specific pseudosubstrates. For α-mannosidase activity, 50-100 µl of tissue homogenate was added to 50 µl of 10 mM p-nitrophenyl-α-D-mannopyranoside (Sigma, St. Louis, MO, USA) in 0.2 M sodium citrate pH 4.2 including 0.4% BSA and 0.08% sodium azide. To test β-hexosaminidase activity, 10 µl of tissue homogenates (diluted between 1:5 and 1:20) were added to 10 mM p-nitrophenyl-N-acetyl-β-D-glucosaminide (Sigma) in 0.1 M sodium citrate pH 4.6, including 0.2% BSA and 0.04% sodium azide in a final volume of 200 µl, and the reaction was performed at 37°C for 16 h (α-mannosidase) or 2 h (β-hexosaminidase). The reaction was stopped by adding 1 ml of 0.4 M glycine in NaOH (pH 10.4). 300 µl of the reaction was used to estimate the absorbance of liberated p-nitrophenol at 405 nm using an Infinite 200 microplate reader (Tecan). The β-hexosaminidase activity of the lysosome-enriched fraction from brain was determined using 25 µl of the Percoll fractions. The assay was performed as described above and incubated for 30 min at 37°C.

### Immunoblotting

Immunoblotting was performed under standard conditions using 4-20% precast SDS gels (Bio-Rad) and PVDF membranes (Merck, Darmstadt, Germany). After incubation with primary antibodies overnight [cathepsin D: 1:500 (Claussen et al., 1997); Lamp1 (clone 1D4B): 1:250 Developmental Studies Hybridoma Bank (University of Iowa, IA, USA), GAPDH: 1:250 (sc-25778, lot #H0612, Santa Cruz Biotechnology, Dallas, TX, USA); Hsp70: 1:1000 (Synaptic systems, cat. no. 149011, kind gift from Prof. Dr Fischer von Mollard, Biochemistry III, Bielefeld University, Bielefeld, Germany); GFAP-glial fibrillary acidic protein (1:2000; clone G-A-5, G3893, Sigma)] and washing, membranes were incubated for 1 h with the appropriate secondary antibody conjugated to horseradish peroxidase (1:5000, Invitrogen, Carlsbad, CA, USA).

### Immunofluorescence

Methods for tissue fixation, preparation of free-floating sections using a Leica 9000 s microtome (Leica, Wetzlar, Germany) and subsequent immunofluorescence staining were performed as described previously (Kowalewski et al., 2015). Primary antibodies used for immunofluorescence were against GFAP (1:500; clone G-A-5, G3893, Sigma); NeuN (1:2000; clone A60, MAB377, Millipore, Merck, Darmstadt, Germany), Olig2 (1:1000; AB9610, Millipore), Iba1 (1:500; GTX100042, GeneTex, Irvine, CA, USA), CD68 (1:500; clone FA-11 (MCA1957, AbD Serotec), calbindin (1:500; D-28K clone CB-955, C9848, Sigma), myelin basic protein (1:1000; MAB386, Millipore), Lamp1 (1:500; clone 1D4B, see above). The monoclonal antibody against GM2 gangliosides (IgM from mouse) was a kind gift from Prof. Kostantin Dobrenis (Albert Einstein College of Medicine of Yeshiva University, NY). Secondary antibodies were Alexa-Fluor conjugates (1:2000) and purchased from Invitrogen. Nuclei were stained with DAPI (Sigma). Confocal microscopy was performed with an LSM 700 (Zeiss, Oberkochen, Germany) or Olympus FV1000 microscope (Tokyo, Japan).

### Filipin staining

Filipin staining was performed on free-floating sections of PFA-fixed mouse brains. Sections were permeabilized with 1% BSA, 0.15% glycine and 0.02% saponin in PBS for 1.5 h. Unesterified cholesterol was stained using 0.005% filipin from a stock solution of 0.1% filipin (Sigma) in 10% DMSO. The sections were washed three times with permeabilization solution, followed by three washes with PBS and were finally mounted with Mowiol and Dabco, and analyzed with a Leica DM5000B epi-fluorescence microscope (excitation filter BP340-380; dichromatic mirror 400; suppression filter LP425).

### Sudan Black B staining

1 g of Sudan Black B (Serva Feinbiochemica, Heidelberg) was resuspended in 100 ml 70% ethanol by boiling. The solution was immediately used after cooling down and filtration. The staining was performed on free-floating sections of PFA-fixed mouse brains. The sections were treated with 50% ethanol for 10 min, covered with the Sudan Black B solution for 5 min and washed three times with 70% ethanol. Slices were incubated in water for 10 min and counterstained with Nuclear Fast Red (Roth, Karlsruhe) for 5 min and finally washed three times with water. The slices were mounted with Mowiol and imaged using a Leica DM IRB microscope.

### Histological examination

Methods for light- and electron-microscopy analysis of mouse organs were performed as described previously (Stinchi et al., 1999). Briefly, mice were deeply anaesthetized with an intraperitoneal injection of ketamine and xylazine. After pre-perfusion with 1% procaine in 0.1 M PBS, fixation was achieved with transcardial vascular perfusion with 6% glutaraldehyde in 0.1 M PBS. Tissue blocks were post-fixed with 2% osmium tetroxide and embedded in araldite. Semi-thin sections were stained with Toluidine Blue. Ultrathin sections were processed with uranyl acetate and lead citrate and viewed with a Zeiss EM 900 electron microscope (Zeiss).

### Isolation and analysis of neutral oligosaccharides

Neutral oligosaccharides were freshly isolated from mouse tissues as described elsewhere (Stinchi et al., 1999). Dried oligosaccharides were diluted in 1 µl H_2_O/mg fresh weight. 10 µl (for analytical TLC) or 25 µl (for preparative TLC) were spotted on silica gel 60 plates (Merck) using the TLC spotter Linomat 5 (CAMAG, Muttenz, Switzerland) and separated by performing TLC for 16 h using 75% acetic acid in H_2_O as the solvent system. The TLC plate was dried, and oligosaccharides were detected by subsequent staining with 0.2% orcinol in 20% sulfuric acid in H_2_O at 120°C for 5 min. For preparative separation, the TLC was performed in duplicate with one plate stained to obtain retention values of single bands and the second undeveloped TLC plate was used to scrape off separated compounds for analysis. For mass spectrometry analysis, the compounds were dissolved from the silica material by adding 100 µl H_2_O and incubating for 30 min at room temperature.

### Analyses of storage material

Storage material was prepared for mass spectrometry analysis as previously described (Damme et al., 2015). Briefly urine samples and water extracts of TLC-isolated compounds were centrifuged at 14,000 ***g*** for 20 min, and 10 µl of extract were mixed with 10 µl of a 0.01 nmol/µl maltotriose internal standard. The mixture was dried in an Eppendorf concentrator and re-dissolved in 10 µl derivatization buffer [64 mg/ml sodium-cyanoborohydride (Sigma) and 41 mg/ml 2-aminobenzamide (Sigma) dissolved in a mixture of dimethylsulfoxide and acetic acid (v/v 7:3) and incubated for 3 h at 70°C followed by evaporation to dryness]. After drying, the sample was re-dissolved in 50 µl acetonitrile with 0.1% trifluoroacetic acid and analyzed with hydrophilic interaction chromatography (HILIC) using a Dionex Ultimate 3000 HPLC system coupled to a Q-Exactive Orbitrap mass spectrometer (Thermo Scientific).

The oligosaccharides were separated on a 100×100 µm ID column packed with TSK-gel Amide-80 column material (Tosoh Bioscience LLC, PA, USA) using a 5-min gradient from 95% to 86% organic solvent (100% acetonitrile with 0.1% trifluoroacetic acid) followed by a 30 min gradient from 86% to 70% organic solvent at a flow rate of 900 nl/min.

The 2-AB-maltodextrose (2-AB)-derivatized internal standard and the storage material of unknown structure were quantified by correlating the peak area obtained from the 2-AB-derivatized internal standard (*m*/*z* 625.24) with peak areas of the detected species of unknown concentrations. The identities of the unknown storage material were based on molecular weights and fragmentation patterns as determined by electrospray ionization tandem mass spectrometry.

### Behavioral analysis

Mice underwent a battery of neuromotor tests, essentially as described previously (Stroobants et al., 2008). Behavioral assessment was initiated at 3 months of age in 15 wild-type and 15 knockout mice (all females). A subset of these knockout animals was retested at 7 months of age (*n*=8, in comparison to 10 wild-type animals, all females) to evaluate progression of motor impairment. Forelimb grip strength was evaluated by letting mice spontaneously grab a T-shaped bar. Connection of the bar to a digital dynamometer (Ugo Basile, Comerio, Italy) allowed quantification of strength, which was averaged over ten trials. Difficulties in proper grabbing of the bar (both forepaws on top) were observed in a subset of animals by an observer that was blind to the conditions. Hence, the presence of recurrent misplacements of the paws was scored (0-1) as an indication of reduced fine motor skills. Home cage activity was recorded in 20×30 cm transparent cages, placed between three infrared beams. The total number of beam crossings was recorded during a 23-h period to evaluate circadian rhythm. The rotarod test was included as a measure of motor coordination and equilibrium. Animals first received 2 min of training at a fixed speed of 4 rpm on the apparatus (MED Associates Inc., St. Albans, Vermont, USA). Following training, four (I-IV) test trials (10 min inter-trial interval) were conducted with an accelerating rotation from 4 to 40 rpm during a 5-min period. Drop latency was registered up to the 5 min cut-off.

Cognition assessment at the age of 7 months included fear conditioning followed by an evaluation of contextual and cued fear memory. This protocol was performed in a plexiglass test chamber (26×22×18 cm), containing a grid floor connected to a constant current shocker (MED Associates Inc., St. Albans, Vermont, USA) (Ahmed et al., 2014). The test set-up was placed inside a sound-proof chamber. On the first day of the experiment, animals received 5 min of habituation time in the apparatus. On the second day, after 2 min of exploration (baseline score), a buzzer was sounded for 30 s. This conditional stimulus was followed by a 2 s foot shock (0.3 mA), the unconditional stimulus. After 1 min exploration, they received a second conditional stimulus and unconditional stimulus pairing, followed by a final minute of exploration (fear conditioning phase). On the third day, animals were placed in the same context for 5 min (contextual fear assessment). After 90 min, the mouse was again placed in the test chamber. Environmental and contextual cues were changed (texture, light and odor). After 3 min of free exploration (pre-cue phase), the auditory stimulus was delivered for 3 min (cued fear assessment). Freezing behavior was recorded every 10 s during each trial block using the standard interval sampling procedure. All procedures and experiments in mice were performed in accordance with local guidelines and were approved by local authorities.

### Statistical methods

Western blots and enzyme activities (mean±s.d.) were analyzed using two-tailed *t*-test (Microsoft Excel, Microsoft Corporation Redmont, WA, USA). qPCR data were analyzed by unpaired *t*-test using GraphPad QuickCalcs (GraphPad Software, La Jolla, CA, USA). Behavioral data were analyzed using SPSS statistics 20 (IBM, Armonk, NY, USA). In behavioral analyses, statistical comparisons of Fuca1^−/−^ mice and wild-type controls were executed using *t*-tests for single genotype effects (grip strength, different phases of fear conditioning) or repeated-measures ANOVA when performance was analyzed over time (cage activity) or trials (rotarod). Sources of variation for cage activity therefore were time of day and genotype, whereas in rotarod tests, trial and genotype were sources of variation.
